# Arterial Blood Gas Analysis in Breath-Hold Divers at Depth

**DOI:** 10.3389/fphys.2018.01558

**Published:** 2018-11-05

**Authors:** Gerardo Bosco, Alex Rizzato, Luca Martani, Simone Schiavo, Ennio Talamonti, Giacomo Garetto, Matteo Paganini, Enrico M. Camporesi, Richard E. Moon

**Affiliations:** ^1^Environmental Physiology and Medicine Laboratory, Department of Biomedical Sciences, University of Padova, Padova, Italy; ^2^ATIP Center for Hyperbaric Medicine, Padova, Italy; ^3^Center for Hyperbaric Medicine and Environmental Physiology, Department of Anesthesiology, Duke University Medical Center, Durham, NC, United States

**Keywords:** arterial blood gas, blood gas analysis, breath-hold diving, physiology, underwater

## Abstract

The present study aimed to evaluate the partial pressure of arterial blood gases in breath-hold divers performing a submersion at 40 m. Eight breath-hold divers were enrolled for the trials held at “Y-40 THE DEEP JOY” pool (Montegrotto Terme, Padova, Italy). Prior to submersion, an arterial cannula in the radial artery of the non-dominant limb was positioned. All divers performed a sled-assisted breath-hold dive to 40 m. Three blood samplings occurred: at 10 min prior to submersion, at 40 m depth, and within 2 min after diver’s surfacing and after resuming normal ventilation. Blood samples were analyzed immediately on site. Six subjects completed the experiment, without diving-related problems. The theoretically predicted hyperoxia at the bottom was observed in 4 divers out of 6, while the other 2 experienced a reduction in the partial pressure of oxygen (paO_2_) at the bottom. There were no significant increases in arterial partial pressure of carbon dioxide (paCO_2_) at the end of descent in 4 of 6 divers, while in 2 divers paCO_2_ decreased. Arterial mean pH and mean bicarbonate (${\mathrm{HCO}}_{3}^{-}$) levels exhibited minor changes. There was a statistically significant increase in mean arterial lactate level after the exercise. Ours was the first attempt to verify real changes in blood gases at a depth of 40 m during a breath-hold descent in free-divers. We demonstrated that, at depth, relative hypoxemia can occur, presumably caused by lung compression. Also, hypercapnia exists at depth, to a lesser degree than would be expected from calculations, presumably because of pre-dive hyperventilation and carbon dioxide distribution in blood and tissues.

## Introduction

Diving with voluntary breath holding (BHD) has been recorded since ancient times and nowadays involves sport competitions, recreational activities, fishing and military operations. Sport breath-hold competitions are classified as follows:

*Constant weight*: swimming with fins to maximum depth and returning to the surface unassisted.
*Variable weight:* divers descend on a weighted sled guided by a vertical rope, and swim back to the surface.
*Free immersion:* pulling oneself down a rope to maximum depth and back by hand only, without using fins or any other equipment.
*No-limits:* riding a weighted sled on descent, and releasing an inflated lift bag or float at the bottom for ascent.
*Static apnea:* breath-hold on air in a shallow swimming pool with the body and face immersed.
*Dynamic apnea:* swimming a horizontal distance on a single breath of air while submerged, either with or without fins (Fitz-Clarke, 2018).

Elite participants have survived dives while breath holding to extraordinary depths of 237 m (women) and 300 m (men), (AIDA — International Association for the Development of Apnea).

In BHD two main factors affect the physiological changes of the human body: the time of breath suspension and the depth of submersion (Bosco et al., 2007, 2018). In the past, regulation of blood flow to tissues and human metabolism during apnea, such as bradycardia induced by this condition, has been extensively studied (Ferrigno and Lundgren, 1999). The diving reflex (DR) is a primordial oxygen-conserving response most studied in large aquatic mammals but observed in all air-breathing vertebrates (Vega, 2017). When a freediver submerges, the main source of oxygen is lost and intrinsic oxygen stores (i.e., pulmonary gas, blood hemoglobin, and muscle myoglobin) are used to sustain aerobic metabolism. However, the diver (human or animal) reaches the aerobic dive limit when these stores are consumed, at which point blood lactate concentration increases (Panneton, 2013). Hypoxic and thermal stimuli lead to a physiological reaction to conserve oxygen. Diving bradycardia, related to apnea duration (Bosco et al., 2007), is the first defensive mechanism, which consists of a vagally induced reduction of heart rate due to vasoconstriction (Lindholm and Lundgren, 2008). Blood flow is selectively directed toward the brain and mediastinal region (i.e., blood shift) by reducing cutaneous, muscular, and splanchnic perfusion (Panneton, 2013). Hemoglobin concentration is increased by splenic contraction, which occurs in the first phase of the sympathetic-response (Baković et al., 2003).

Insight into pulmonary gas exchange has been largely achieved by modeling and measurement of exhaled gas partial pressures (Otis et al., 1948; Schaefer and Carey, 1962; Lanphier and Rahn, 1963; Craig and Harley, 1968; Ferretti et al., 1991;; Lindholm and Lundgren, 2006; Overgaard et al., 2006). A published study reported direct measurements of arterial blood gas tensions during submersed diving at depths of 20 cm (Olsen et al., 1962) and up to 7 m (Qvist et al., 1993). Arterial blood gases have also been measured during simulated dives to 20 m in a hyperbaric chamber (Muth et al., 2003). However, measurements obtained were significantly different from those predicted (Olsen et al., 1962; Qvist et al., 1993; Muth et al., 2003). The increase in ambient pressure (and hence partial pressures of intrapulmonary gases) during descent should induce hyperoxia and hypercapnia. Due to consumption of oxygen during the descent and bottom phases and reduced ambient pressure during ascent, hypoxia is expected during or after surfacing.

The time course of arterial blood gas changes during breath-hold dives did not correspond to a theoretical estimation exclusively based on Dalton’s law of partial pressures. This prediction has not been accurate in the case of partial pressure of carbon dioxide (paCO_2_), (Muth et al., 2003).

Blood solubility of O_2_ is much less than that of (carbon dioxide) CO_2_, thus due to the buffering effect of the blood and tissues, changes in the partial pressure of carbon dioxide (paCO_2_) are attenuated compared with paO_2_. Moreover, the compression-related rise in intrathoracic blood volume (Ferrigno and Lundgren, 1999) associated with increased pulmonary blood further contributes to this buffering effect on paCO_2_. Finally, during ascent an increase in arterial paCO_2_ is blunted by the Haldane effect, that is the increased solubility of CO_2_ in blood at low hemoglobin-oxygen saturation. The initially reduced CO_2_ level (induced by hyperventilation at the surface) facilitates prolongation of the BH time due to reduced drive to breathe, which in turn allows arterial paO_2_ to drop to lower levels, often enough to cause hypoxic syncope during or immediately after ascent (Lindholm and Gennser, 2005).

The present study aimed to evaluate the partial pressure of arterial blood gases in well-trained athletes performing a breath-hold dive in water with arterial blood sampling before, during and after submersion at 40 m. This is a report of the first experiment during submersion at 40 m actual depth in which arterial blood samples have been measured at depth.

## Materials and Methods

### Subjects

Eight well-trained (5 years of high-level experience, with a typical dive duration of 200 s and a maximal depth of 50–60 m for all of them) healthy breath-hold divers were enrolled and medically screened. Inclusion criteria were: to have no history of orthopedic, cardiovascular, renal or metabolic disorders. Exclusion criteria were: allergy to local anesthetics, abnormal coagulation, alterations of the arterial vascularization of the upper limbs or vasculopathies. Finally, six of eight divers (characteristics are reported in Table 1) completed this study. Two of eight divers were excluded due to difficult arterial cannulation.

**Table 1:** Demographic and anthropometric parameters.

Subject	Age (years)	Body mass (kg)	Height (m)	BMI (kg/m^2^)
1	55–60	82	1.71	28.0
2	50–55	80	1.80	24.6
3	45–50	63	1.72	21.2
4	40–45	80	1.81	24.4
5	40–45	94	1.80	29.0
6	30–35	70	1.75	22.8
Mean ±SD	46.6 ±9.3	78.1 ±10.6	1.76 ±0.04	25 ±2.9


*The mean values ± SD, (*n* = 6) for body mass (kg), height (m) and age (years), and Body Mass Index (BMI, kg/m^2^) are indicated.*

### Experimental Design

The experimental protocol received the approval by the Human Ethical Committee (n° HEC-DSB/03-18) of the Department of Biomedical Science of University of Padova and adhered to the principles of the Declaration of Helsinki. Written informed consent was obtained from the divers before enrollment in the study.

This was an observational experimental design, with repeated measures conducted under three different experimental conditions. Fourteen days before the beginning of the testing sessions, a familiarization meeting was organized to ensure that all the divers knew the protocol and could complete the scheduled program. In particular, the divers were instructed about the experimental procedures in order to guarantee their correct execution.

All divers, accompanied by a professional instructor, performed a sled-assisted breath-hold dive to 40 m. Prior to submersion, an arterial cannula was inserted in the radial artery of the non-dominant limb. Procedures for arterial access and hematic collection are explained below. To ensure the safety of the divers a second professional diver was stationed during the descending and ascending phases at 20 m depth. Two medical assistants were employed in the blood sampling procedures at 40 m. Finally, two additional medical assistants obtained the last arterial sample after surfacing. All the medical assistants were three anesthesiologists, an interventional cardiologist, an emergency physician and internal medicine physician.

Training procedures were standardized before the experimental dive. In particular, all the divers prepared themselves with two breath-hold dives after the first sampling: one to 10 m and the other to 12 m. Furthermore, all the divers performed hyperventilation (not forced) for 2 min prior to submersion. The experimental setting for the trials was the world’s deepest pool “Y-40 THE DEEP JOY” with a water temperature of 31.5±0.5°C located in Montegrotto Terme (Padova, Italy).

### Arterial Access and Hematic Collection

The positioning of the radial arterial access occurred on the non-dominant limb, only after confirmation of the radial and ulnar arterial patency via the Allen test (Fuhrman et al., 1992), using the following procedure:

(1) topical anesthesia with ice or Emla^®^;
(2) local anesthesia with 1% lidocaine;
(3) skin asepsis;
(4) Percutaneous cannulation with 20G (3 Fr) Teflon catheter, with “catheter over – the – needle” technique.

If objective or subjective anomalies were found, the catheter was immediately removed and the most appropriate action evaluated.

After successful positioning, the arterial catheter was fixed to the skin by means of a special adhesive band (3M^TM^Tegaderm^TM^ I.V. Advanced Securement Dressing 10 cm × 12 cm, 3M Company, Maplewood, MN, United States), and then connected to a circuit with Luer Lok-type fittings to prevent leakage, assembled in a proximal-distal order by:

– one rigid plastic connection, single lumen, 10 cm in length;
– one lockable three-way stopcock;
– one 2.5 ml heparinized glass syringe for standard blood gas sampling, on the first stopcock;
– one 10 ml plastic syringe filled with 3 ml of 0.9% NaCl solution, on the second stopcock, used both for arterial blood aspiration before sampling (“dead space”) and for flushing and wash-out of the arterial line after the sample collection.

**FIGURE 1:**
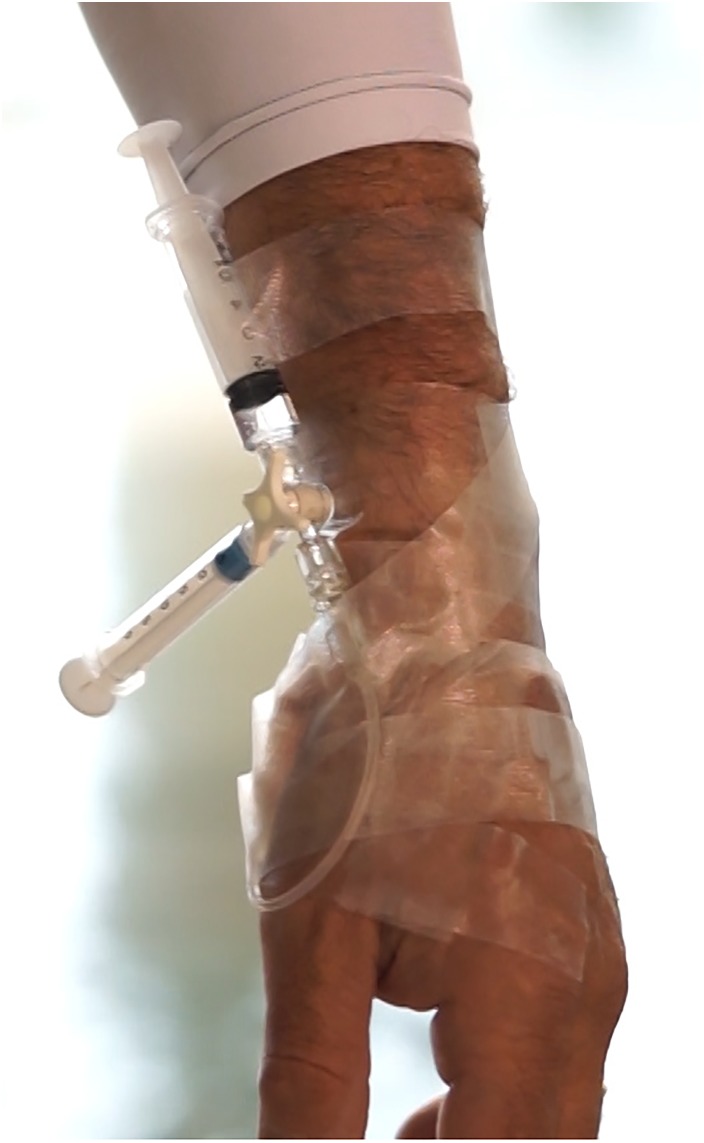
Photo detail of the circuit inserted in the radial artery of a diver described in the text.

The circuit (Figure 1) was filled with 0.9% NaCl solution and gas bubbles carefully removed. In case of disconnection, the subject was trained to turn the stopcock to prevent bleeding.

Blood collection at depth consisted of the following steps:

(1) Opening of second stopcock;
(2) Slow aspiration of at least 5 ml of arterial blood with the plastic syringe;
(3) Holding the plunger of the syringe firmly, rotation of the three-way stopcock to open the first stopcock;
(4) With the first channel opened, slow aspiration of at least 2 ml of arterial blood into the heparinized syringe;
(5) Holding the plunger of the syringe firmly, rotation of the three-way stopcock to close the first channel and open the second, to flush the circuit;
(6) Closing all the channels with a 45°-intermediate position of the lockable three-way stopcock.

**FIGURE 2:**
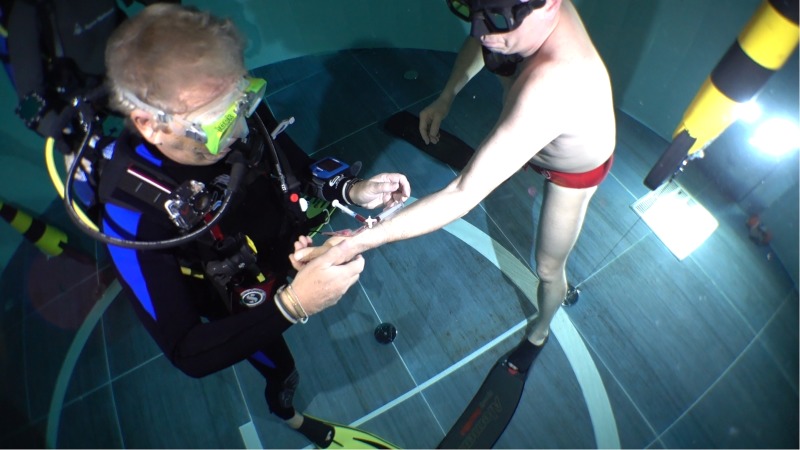
A medical assistant (anesthesiologist) drawing arterial blood samples from a BH divers at the depth of 40 m. Written informed consent was obtained from the individual for the publication of this image.

Blood sampling occurred as follows:

(1) Before the dive: 10 min prior to submersion.
(2) At 40 meters depth (underwater sampling): 5 s after the end of the descent (Figure 2).
(3) At the end of the dive: within 2 min after diver’s surfacing and after resuming normal ventilation.

The circuit remained intact until the diver surfaced. Once the subject left the water, the syringe was separated from the circuit, maintained on ice for at least 2 min, and analyzed in the blood gas analyzer present on site (*i-STAT Alinity system*, Abbott Diagnostics, IL, United States) obtaining data on pH, paCO_2_, arterial oxygen Saturation (SaO_2_ %), paO_2_, bicarbonate (${\mathrm{HCO}}_{3}^{-}$), oxygen-saturated hemoglobin Hb-O_2_ saturation, base excess of extracellular fluid (BE-ecf), total carbon dioxide (tCO_2_), and lactate. Timing of the descent, time on the bottom and ascent was recorded. In air tight glass syringes, blood gas measurements from normal individuals are stable for more than 30 min at room temperature (Knowles et al., 2006) and several hours if maintained on ice.

At the end of the experiment, the arterial cannula was removed under aseptic conditions and a compression bandage applied for 2 h. The insertion area was monitored over the next 2 days.

### Statistical Analysis

Kolmogorov-Smirnov test was employed to check data normality distribution. One-way ANOVA for repeated measures was performed to investigate dependent variables in the three different time-points. Then, pairwise comparisons Tukey-Kramer HSD *post hoc* testing was used. Data analysis was performed using the software JMP Pro 13.1 (SAS Institute, Cary, NC, United States). Data are presented as mean ± standard deviation (SD). Significant level for differences was set to *p* < 0.05.

## Results

All subjects completed the experiment with no neurological symptoms, LOC or evidence of pulmonary barotrauma. Descent time was typically 45.16 ±2.78s and arterial sample at depth was obtained immediately after (see Figure 1). Divers spent 39–52 s at maximum depth for sampling, then the ascent phase took between 34 and 47 s. Once at the surface, each diver swam to the poolside. The third arterial blood sample was obtained within 2 min after surfacing and restoring of breathing. Individual times are shown in Table 2.

**Table 2:** Breath-hold diving (BHD) duration.

Subject	Starting time	Ending time	BHD duration (s)	Descent time	Bottom time	Ascent Time
1	18:37:48	18:39:57	129	42	52	35
2	18:49:00	18:51:25	145	47	51	47
3	18:33:37	18:35:50	133	46	49	38
4	19:56:45	19:58:50	125	45	44	36
5	19:52:50	19:55:08	138	49	46	43
6	20:01:45	20:03:40	115	42	39	34
Mean			130.8	45.1	46.8	38.8
*SD*			10.4	2.7	4.8	5.1


*Table provides the specific time-profile during all the phases of the human exposure at 40-m depth.*

Individual blood gas results are shown in Table 3. The theoretically predicted hyperoxia at the bottom was observed in only 4 divers out of 6: in subjects No 3 and 5 there was a reduction in pO_2_ at the bottom (No. 3: 89 → 75 mmHg; No. 5: 97 → 61 mmHg), while the other divers showed a mean increase of 181% in their arterial paO_2_. Subject No. 5 experienced mild hypoxemia: after the ascent, measured arterial paO_2_ was 53 mmHg and O_2_ saturation was 88%.

**Table 3:** Arterial blood gas results.

	paO_2_ (mmHg)			SaO_2_ (%)			paCO_2_ (mmHg)			pH		
Subject	Pre-Dive	40 m	Post-Dive	Pre-Dive	40 m	Post Dive	Pre-Dive	40 m	Post Dive	Pre-Dive	40 m	Post Dive
1	92	290	73	98	100	95	34.0	44.3	33.8	7.50	7.30	7.46
2	101	218	67	98	100	93	37.4	31.9	37.3	7.46	7.45	7.42
3	89	75	93	97	94	98	38.1	46.5	35.7	7.43	7.36	7.43
4	86	261	102	97	100	98	43.3	49.3	39.3	7.42	7.38	7.48
5	97	61	53	98	94	88	35.2	27.4	35.8	7.44	7.51	7.43
6	97	282	79	98	100	96	38.3	56.8	39.8	7.43	7.30	7.40
Mean	93.7	197.8^∗^	77.8^†^	97.6	98	94.6	37.7	42.7	36.9	7.44	7.38	7.42
*SD*	5.6	103.7	17.7	0.5	3.0	3.7	3.2	12.3	1.9	0.01	0.08	0.02

	**${\mathrm{HCO}}_{3}^{-}$ (mmol/L)**			**BE, ecf (mmol/L)**			**tCO_2_ (mmol/L)**			**Lactate (mmol/L)**		
**Subject**	**Pre-Dive**	**40 m**	**Post-Dive**	**Pre-Dive**	**40 m**	**Post Dive**	**Pre-Dive**	**40 m**	**Post Dive**	**Pre-Dive**	**40 m**	**Post Dive**

1	26.5	26.7	24	3	2	0	28	28	25	0.7	1.8	1.9
2	26.3	22.1	24	2	-2	-1	27	23	25	1.05	1.5	2.7
3	25.3	26.1	23.8	1	1	0	26	28	25	0.9	1.07	2.1
4	27.8	29.4	25.3	3	4	1	29	31	27	0.5	1.1	2.2
5	23.9	21.7	23.7	0	-1	-1	25	22	25	0.4	1.4	2.7
6	25.2	28.2	24.4	1	2	0	26	30	26	0.6	1.05	1.2
Mean	25.8	25.7	24.2	1.6	1	-0.1	26.8	27	25.5	0.7	1.3^¥^	2.1^§^ #
*SD*	1.3	3.1	0.5	1.2	2.1	0.7	1.4	3.6	0.8	0.2	0.3	0.5


*Partial pressure of carbon dioxide (paCO_*2*_), arterial oxygen Saturation (SaO_*2*_ %), partial pressure of oxygen (paCO_*2*_), pH, bicarbonate (HCO_*3*_^-^), base excess of extracellular fluid (BE-ecf), total carbon dioxide (tCO_*2*_), and lactate. ^∗^*P* < 0.05 vs. Pre-Dive; ^†^*P* < 0.05 vs. 40 m; ^§^*P* < 0.001 vs. Pre-Dive; ^#^*P* < 0.001 vs. 40-m; ^¥^*P* < 0.05 vs. 40-m.*

There were variable changes in arterial paCO_2_ after the descent. At 40 m paCO_2_ increased from baseline in 4 of 6 divers, while in two divers paCO_2_ decreased (No. 2: 31.9 mmHg; No. 5: 27.4 mmHg). Arterial mean pH and mean level of ${\mathrm{HCO}}_{3}^{-}$ exhibited minor changes. There was a statistically significant increase in mean arterial lactate level after the exercise (0.71 → 1.35 → 2.15 mmol/L).

## Discussion

**FIGURE 3:**
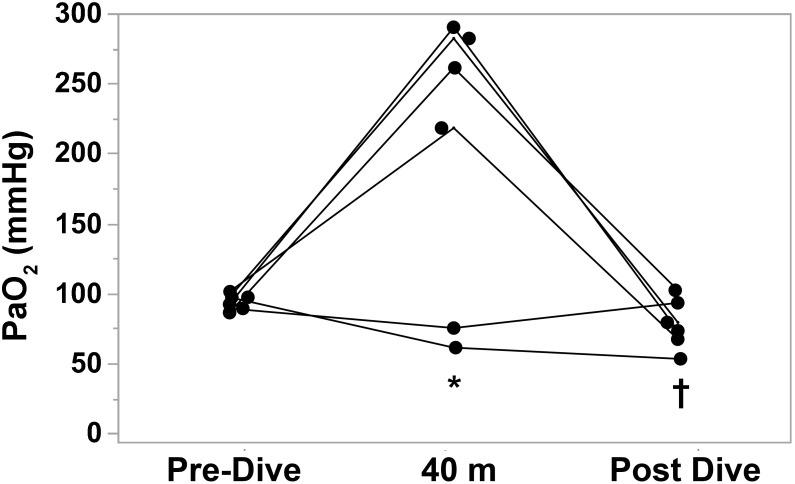
Individual arterial partial pressure of oxygen (paO_2_) measurements before, at depth (40 m), and after BHD. Each subject is compared to his own dive values (*n* = 6). ^∗^*P* < 0.05 vs. Pre-Dive; ^†^*P* < 0.05 vs. 40 m.

This is the first study to evaluate the partial pressure of arterial blood gases in breath-hold divers before, during, and after a breath-hold dive in water at a depth of 40 m. Our measurements are mostly consistent with theoretically expected hyperoxia at the bottom, induced by the rise in ambient pressure. The expected increase in paO_2_ at maximum depth due to the rise in alveolar paO_2_ was observed in 4 of 6 subjects. Surprisingly, in two subjects out of 6 (who had the same training and experience as the others), paO_2_ was lower than predicted, 75 and 61 mmHg, respectively (Figure 3). We hypothesize that in these two subjects the lower than expected paO_2_ was due to ventilation/perfusion mismatch and right-to-left intrapulmonary shunt due to atelectasis caused by the significant reduction in lung volume at 40 m (20% of the pre-dive volume), (Fitz-Clarke, 2018). Blood flow through an atelectatic area would likely be augmented by pulmonary vasodilatation caused by the combination of immersion (Cherry et al., 2009) and pulmonary vascular engorgement due to pressure differential between pulmonary blood vessels and alveolar gas as lung volume during descent approaches residual volume. As to why these two individuals did not become profoundly hypoxic during ascent to the surface, we propose that expansion of atelectatic lung reversed the lung changes and secondary gas exchange abnormalities that led to relative hypoxemia at 40 m. Of note, subjects 2 and 5 both had hypocapnia at maximum depth. Presumably these individuals both hyperventilated immediately prior to the dive, after the initial surface blood sample had been obtained.

We were unable to obtain blood gas samples immediately upon surfacing, thus the post-dive values we report undoubtedly overestimated the paO_2_ at the end of ascent. Nevertheless in 4 of 6 subjects paO_2_ after the dive was lower than pre-dive, and in one subject arterial hemoglobin-oxygen saturation was 88%. Hypoxia of ascent is the most dangerous situation in BHD, causing symptoms that range from LMC to transient cognitive impairment or LOC leading to risk of drowning. These manifestations occur especially in the ascent phase, presumably due to reduced ambient pressure during decompression and reduced alveolar oxygen levels due to consumption. None of our subjects experienced such symptoms. Previous studies reported that elite athletes did not have symptoms if their alveolar oxygen partial pressure was above 25 mmHg (end-tidal O_2_ in exhalations after the dive), (Lindholm and Lundgren, 2006; Overgaard et al., 2006).

Our results revealed a slight increase in arterial paCO_2_ of about 5 mmHg at the bottom in 4 of 6 divers. This rise in paCO_2_ is consistent with the observations of Muth and colleagues, who found that during a simulated BHD to 20 m the arterial pCO_2_ of two elite athletes was increased from 22.5 and 29.2 mmHg before diving to 42.8 and 44.2 mmHg at maximum depth (Muth et al., 2003). Furthermore, our data confirm that gas laws alone do not predict arterial paCO_2_ changes during BHD, as previously suggested by Muth and Liner (Linér et al., 1993; Muth et al., 2003), who hypothesized that humans’ ability to withstand deep and long apneas without severe hypercapnia is mainly due to the storage capacity of the more soluble carbon dioxide (CO_2_) in the blood, thus attenuating the chemoreflex-induced drive to breathe. Moreover, they suggested several physiological mechanisms of adaptation that could explain the deviation of theoretically predicted blood gas values, such as chest volume reduction during descent and the diving-induced increase in pulmonary blood flow which allows a higher uptake of O_2_ from the lungs (Muth et al., 2003). The apparently small increase in paCO_2_ in our data may reflect the fact that our pre-dive blood samples were obtained 10 min before the actual dive, after which the subjects may have hyperventilated to a lower paCO_2_.

The minimal but statistically significant increase in lactate is consistent with anaerobic metabolism due to peripheral vasoconstriction caused by the diving reflex, or conceivably muscle activity during and after ascent.

## Conclusion

To our knowledge ours is the first attempt to verify real changes in blood gases at a depth of 40 m during a breath-hold descent in well-trained free-divers. We have demonstrated that relative hypoxemia can occur at maximum depth, presumably due to pulmonary gas exchange abnormalities caused by lung compression. Hypercapnia exists at depth, to a lesser degree than would be expected from calculation of alveolar paCO_2_, presumably because of carbon dioxide uptake and storage in the blood and tissues.

## Author Contributions

GB, EC, and RM conceived and designed the experiments. GB, AR, LM, SS, ET, GG, and MP performed the experiments. MP, RM, and AR analyzed the data. GB contributed the materials. GB, AR, MP, EC, and RM wrote the paper.

## Conflict of Interest Statement

The authors declare that the research was conducted in the absence of any commercial or financial relationships that could be construed as a potential conflict of interest.

## Abbreviations

BHD: breath-hold diving
LMC: loss of motor control
LOC: loss of consciousness

## References

[B1] Baković D. Valic Z. Eterović D. Vukovic I. Obad A. Marinović-Terzić I. (2003). Spleen volume and blood flow response to repeated breath-hold apneas. *J. Appl. Physiol.* 95 1460–1466. 10.1152/japplphysiol.00221.2003 12819225

[B2] Bosco G. Di Tano G. Zanon V. Fanò G. (2007). Breath-hold diving: a point of view. *Sport Sci. Health* 2:47 10.1007/s11332-007-0038-y

[B3] Bosco G. Rizzato A. Martani L. Schiavo S. Talamonti E. Garetto G. (2018). Arterial Blood Gas Analysis in Breath-Hold Divers at Depth. *Front. Physiol.* 9 1558 10.3389/fphys.2018.01558 PMC6230561 30455649

[B4] Cherry A. D. Forkner I. F. Frederick H. J. Natoli M. J. Schinazi E. A. Longphre J. P. (2009). Predictors of increased PaCO2 during immersed prone exercise at 4.7 ATA. *J. Appl. Physiol.* 106 316–325. 10.1152/japplphysiol.00885.2007 18787095

[B5] Craig A. B. Harley A. D. (1968). Alveolar gas exchanges during breath-hold dives. *J. Appl. Physiol.* 24 182–189. 10.1152/jappl.1968.24.2.182 5637681

[B6] Ferretti G. Costa M. Ferrigno M. Grassi B. Marconi C. Lundgren C. E. (1991). Alveolar gas composition and exchange during deep breath-hold diving and dry breath holds in elite divers. *J. Appl. Physiol.* 70 794–802. 10.1152/jappl.1991.70.2.794 1902459

[B7] Ferrigno M. Lundgren C. (1999). “Human breath-hold diving,” in *The Lung at Depth* ed. Miller J. (New York, NY: Marcel Dekkers Inc.) 576–585.

[B8] Fitz-Clarke J. R. (2018). Breath-hold diving. *Compr. Physiol.* 8 585–630. 10.1002/cphy.c160008 29687909

[B9] Fuhrman T. M. Pippin W. D. Talmage L. A. Reilley T. E. (1992). Evaluation of collateral circulation of the hand. *J. Clin. Monit.* 8 28–32. 10.1007/BF01618084 1538249

[B10] Knowles T. P. Mullin R. A. Hunter J. A. Douce F. H. (2006). Effects of syringe material, sample storage time, and temperature on blood gases and oxygen saturation in arterialized human blood samples. *Respir. Care* 51 732–736. 16800906

[B11] Lanphier E. H. Rahn H. (1963). Alveolar gas exchange during breath-hold diving. *J. Appl. Physiol.* 18 471–477. 10.1152/jappl.1963.18.3.471 31083864

[B12] Lindholm P. Gennser M. (2005). Aggravated hypoxia during breath-holds after prolonged exercise. *Eur. J. Appl. Physiol.* 93 701–707. 10.1007/s00421-004-1242-y 15778900

[B13] Lindholm P. Lundgren C. E. (2008). The physiology and pathophysiology of human breath-hold diving. *J. Appl. Physiol.* 106 284–292. 10.1152/japplphysiol.90991.2008 18974367

[B14] Lindholm P. Lundgren C. E. G. (2006). Alveolar gas composition before and after maximal breath-holds in competitive divers. *Undersea Hyperb. Med.* 33 463–467. 17274316

[B15] Linér M. H. Ferrigno M. Lundgren C. E. (1993). Alveolar gas exchange during simulated breath-hold diving to 20 m. *Undersea Hyperb. Med.* 20 27–38. 8471957

[B16] Muth C. M. Radermacher P. Pittner A. Steinacker J. Schabana R. Hamich S. (2003). Arterial blood gases during diving in elite apnea divers. *Int. J. Sports Med.* 24 104–107. 10.1055/s-2003-38401 12669255

[B17] Olsen C. R. Fanestil D. D. Scholander P. F. (1962). Some effects of apneic underwater diving on blood gases, lactate, and pressure in man. *J. Appl. Physiol.* 17 938–942. 10.1152/jappl.1962.17.6.938 13940068

[B18] Otis A. B. Rahn H. Fenn W. O. (1948). Alveolar gas changes during breath holding. *Am. J. Physiol.* 152 674–686. 10.1152/ajplegacy.1948.152.3.674 18863173

[B19] Overgaard K. Friis S. Pedersen R. B. Lykkeboe G. (2006). Influence of lung volume, glossopharyngeal inhalation and P(ET) O2 and P(ET) CO2 on apnea performance in trained breath-hold divers. *Eur. J. Appl. Physiol.* 97 158–164. 10.1007/s00421-006-0156-2 16525813

[B20] Panneton W. M. (2013). The mammalian diving response: an enigmatic reflex to preserve life? *Physiology* 28 284–297. 10.1152/physiol.00020.2013 23997188 PMC3768097

[B21] Qvist J. Hurford W. E. Park Y. S. Radermacher P. Falke K. J. Ahn D. W. (1993). Arterial blood gas tensions during breath-hold diving in the Korean ama. *J. Appl. Physiol.* 75 285–293. 10.1152/jappl.1993.75.1.285 8376276

[B22] Schaefer K. E. Carey C. R. (1962). Alveolar pathways during 90-foot, breath-hold dives. *Science* 137 1051–1052. 10.1126/science.137.3535.1051 14497944

[B23] Vega J. L. (2017). Edmund Goodwyn and the first description of diving bradycardia. *J. Appl. Physiol.* 123 275–277. 10.1152/japplphysiol.00221.2017 28495845

